# Impact of Metformin on Endothelial Ischemia-Reperfusion Injury in Humans In Vivo: A Prospective Randomized Open, Blinded-Endpoint Study

**DOI:** 10.1371/journal.pone.0096062

**Published:** 2014-04-22

**Authors:** Saloua El Messaoudi, Tim H. Schreuder, Roel D. Kengen, Gerard A. Rongen, Petra H. van den Broek, Dick H. J. Thijssen, Niels P. Riksen

**Affiliations:** 1 Department of Pharmacology-Toxicology, Radboud University Medical Center, Nijmegen, The Netherlands; 2 Department of Cardiology, Radboud University Medical Center, Nijmegen, The Netherlands; 3 Department of Physiology, Radboud University Medical Center, Nijmegen, The Netherlands; 4 Department of General Internal Medicine, Radboud University Medical Center, Nijmegen, The Netherlands; 5 Research Institute for Sports and Exercise Sciences, Liverpool John Moores University, Liverpool, United Kingdom; Kurume University School of Medicine, Japan

## Abstract

**Introduction:**

Large prospective studies in patients with type 2 diabetes mellitus have demonstrated that metformin treatment improves cardiovascular prognosis, independent of glycemic control. Administration of metformin potently limits infarct size in murine models of myocardial infarction. This study examined, for the first time in humans, whether metformin limits ischemia-reperfusion (IR) injury *in vivo* using a well-validated forearm model of endothelial IR-injury.

**Methods:**

Twenty-eight healthy volunteers (age 41±6 years, 10 male/16 female) were randomized between pretreatment with metformin (500 mg three times a day for 3 days) or no treatment in a Prospective Randomized Open Blinded Endpoint study. Brachial artery flow mediated dilation (FMD) was measured before and after 20 minutes of forearm ischemia and 20 minutes of reperfusion. FMD analysis was performed offline by investigators blinded for the treatment arm.

**Results:**

Baseline FMD did not differ between metformin pretreatment and no pretreatment (6.9±3.6% and 6.1±3.5%, respectively, p = 0.27, n = 26). FMD was significantly lower after forearm IR in both treatment arms (4.4±3.3% and 4.3±2.8%, respectively, P<0.001 in both conditions). A linear mixed model analysis revealed that metformin treatment did not prevent the decrease in FMD by IR.

**Conclusion:**

A 3 day treatment with metformin in healthy, middle-aged subjects does not protect against endothelial IR-injury, measured with brachial artery FMD after forearm ischemia. Further studies are needed to clarify what mechanism underlies the cardiovascular benefit of metformin treatment.

**Trial Registration:**

ClinicalTrials.gov NCT01610401

## Introduction

Despite optimal reperfusion strategies, morbidity and mortality remain significant in patients suffering an acute myocardial infarction. Therefore, much effort is put into developing novel strategies to limit ischemia-reperfusion (IR) injury.[1] Interestingly, large observational and intervention studies have shown that overall cardiovascular mortality is lower in patients with type 2 diabetes mellitus who are treated with metformin, than in patients treated with alternative glucose-lowering drugs, despite similar glycemic control.[2]–[6] This observation suggests that metformin has direct cardioprotective effects.[7] Indeed, in murine models of myocardial infarction, performed in diabetic as well as in non-diabetic animals, administration of metformin limits myocardial infarct size.[8]–[11] This cardioprotective effect is mediated by activation of adenosine monophosphate activated protein kinase (AMPK) and adenosine receptor stimulation.[9], [10] Whether metformin treatment also directly protects against IR injury in humans is currently unknown.

Myocardial IR also induces endothelial dysfunction, causing endothelial swelling and impaired endothelium-dependent relaxation, which can further impede proper tissue reperfusion.[12] Several strategies, including ischemic preconditioning and postconditioning have been reported to limit IR-induced endothelial dysfunction in healthy humans [13], [14].

In this study, we investigate for the first time in humans whether metformin limits endothelial IR injury *in vivo* by measuring flow mediated dilation (FMD) of the brachial artery before and after prolonged ischemia and reperfusion of the forearm. We test the hypothesis that short-term pretreatment with metformin limits endothelial IR injury in humans *in vivo*.

## Methods

### Ethics Statement

The protocol is approved by the Institutional Review Board of the Radboud University Medical Centre, and was performed in the Radboud University Medical Centre in compliance with the recommendations of the Declaration of Helsinki. All patients signed for informed consent before participation. The study is registered at www.clinicaltrials.gov (NCT01610401). The authors confirm that all ongoing and related trials for this drug/intervention are registered. The protocol for this trial and supporting CONSORT checklist are available as supporting information; see Checklist S1 and Protocol S1.

### Participants

**Figure 1:**
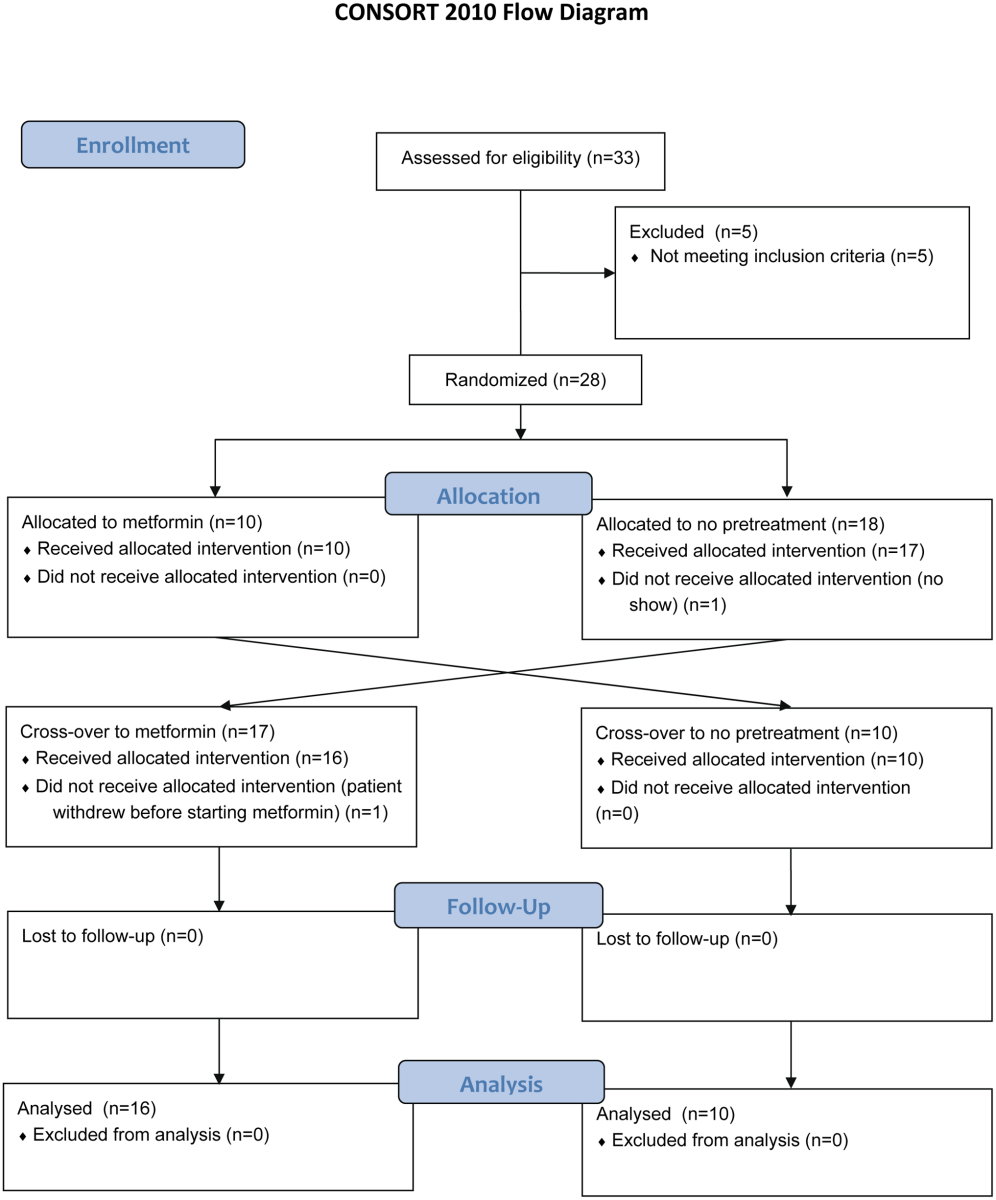
Consort 2012 flow diagram of the study.

We included 28 healthy, non-smoking adult volunteers in this study. All subjects were free of cardiovascular disease, diabetes mellitus, hypertension (systolic blood pressure ≥140 and/or diastolic ≥90 mm Hg) and hypercholesterolemia (random total cholesterol>6.5 mmol/L). We also excluded professional athletes and those who were taking concomitant medication. Oral contraceptive use by female participants was permitted and these females were asked to continue their contraceptive throughout the study to maintain stable hormone levels. Females not on oral contraceptives were measured at identical times in their menstrual cycle, to exclude any confounding effects of circulating hormones on endothelial function.[15], [16] Two participants withdrew during the study. Therefore, 26 subjects finished the trial protocol (Figure 1).

### Experimental Design

In a prospective randomized open blinded end-point (PROBE) study, subjects were allocated to treatment with either metformin 500 mg (Mylan, Bunschoten, The Netherlands) three times a day for 3 days, to ensure a steady state plasma concentration, or no pretreatment (figure 1). Simple random allocation was performed by an independent researcher by dice-throwing for each individual patient (even number: starting with metformin; uneven number: starting with no treatment). The last dose of metformin was given approximately 3 hours before the experiments. Subjects attended our laboratory twice, separated by at least 14 days to prevent any cross-over effect of metformin. Brachial artery endothelial function was measured with flow-mediated dilation (FMD) in the right arm, before and after 20 minutes of forearm ischemia. Forearm ischemia was induced by inflating a pneumatic cuff around the upper arm for 20 minutes and this was followed by 20 minutes of reperfusion. Patient recruitment was performed between May 15^th^ 2012 and October 4^th^ 2012. The first patient was included on May 15^th^ 2012 and the last visit of the last patient was November 20^th^ 2012.

### Measurements

All subjects had to abstain from caffeine consumption and strenuous exercise for at least 24 hours before the measurement. Measurements were performed after an overnight fast of at least 6 hours. Before the test, venous blood was taken to assess metformin and caffeine levels. After removing phospholipids and proteins with HybridSPE-phospholipids columns (Supelco), the plasma metformin concentration was determined with LC-MS/MS, using an Accela U-HPLC (Thermo Fischer Scientific) coupled to a TSQ Vantage (Thermo Fisher Scientific) triple quadropole mass spectrometer. The compounds were separated on a Zorbax HILIC Plus (100×2.1 mm, 3.5 µm particle size; Agilent Technologies). As internal standard we used metformin-d6 (Toronto Research Chemicals Inc.) The elution gradient was as follows: 0 min, 100% B; 5 min, 50% B; and 6 min, 100% B. Solvent A consisted of 2 mM NH4format+0.1% formic acid in H2O and Solvent B consisted of 2 mM NH4format+0.1% formic acid in 90% Acetonitril. The column temperature was set at 40°C, and the flow rate was 200 µl/min. The effluent from the U-HPLC was passed directly into the electrospray ion source. Positive electrospray ionization was achieved using a nitrogen sheath gas with ionization voltage at 3500 Volt. The capillary temperature was set at 290°C. Detection of metformin and the internal standard was based on isolation of the protonated molecular ion, [M+H]+ and subsequent MS/MS fragmentations and a selected reaction monitoring (SRM) were carried out. The following SRM transitions were used: for metformin *m/z* 130,1(parent ion) to *m/z* 60,1 and 71,1 (both product ions) and for d6-metformin m/z 136,1 (parent ion) to m/z 77,1 (product ion).

Plasma caffeine concentrations were determined by use of reversed phase HPLC with UV detection set at 273 nm, as previously described [17].

#### Flow Mediated Dilation (FMD)

Endothelium-dependent vasodilation was assessed using FMD according to recent guidelines.[18] Participants rested in the supine position in a temperature-controlled room (22°C) for at least 15 minutes to allow baseline assessment of heart rate and blood flow. The subjects were tested at the same time of day to prevent diurnal variation in FMD responses. Mean arterial pressure was determined using a manual sphygmomanometer placed around the left arm.

To examine brachial artery FMD, the right arm was extended and positioned at an angle of ∼80° abduction from the torso. A rapid inflation and deflation pneumatic cuff (D.E. Hokanson, Bellevue, WA) was placed distal to the olecranon process to provide an ischemic stimulus distal from the brachial artery. A 10-MHz (T3000, Terason, Aloka, UK) multi-frequency linear array probe attached to a high-resolution ultrasound machine was used to perform imaging. The brachial artery was imaged in the distal third of the upper arm. Ultrasound parameters were set to optimize longitudinal B-mode images of the lumen/arterial wall interface. A continuous Doppler velocity assessment was obtained simultaneously, and data were collected using the lowest possible insonation angle (always <60°), which did not vary during each study. [18] After a resting period of at least 15 minutes, 1 minute of baseline recording of the arterial diameter and velocity was performed. Subsequently, the occlusion cuff was inflated to 220 mmHg for 5 minutes. The arterial diameter and velocity recordings were restarted at least 30 seconds before cuff deflation and continued for at least 3 minutes after deflation. Peak arterial diameter and flow, and the time to reach this peak after cuff deflation, were recorded. Subsequently, the rapid inflation/deflation pneumatic cuff was positioned proximally around the upper arm to provide an occlusion for 20 minutes, so that the brachial artery was within the ischemic zone and was exposed to IR. The cuff was inflated for 20 minutes to 220 mmHg, which was followed by 20 minutes of reperfusion. Finally, the FMD measurement was repeated 20 minutes after reperfusion. All measurements were performed by the same, well-experienced sonographer who was blinded for the treatment allocation.

### Brachial Artery Diameter and Blood Flow Analysis

Analysis of the brachial artery diameter was performed by an investigator who was blinded for the experimental treatment, using custom-designed edge-detection and wall-tracking software, which is independent of investigator bias.[19] Baseline data were calculated across the 1 minute preceding cuff inflation. Following cuff deflation, peak diameter was automatically detected according to an algorithm as described in detail elsewhere.[20] Within-subject reproducibility of the FMD using this semi-automated software is 6.7–10.5% (coefficient of variation).[21] Post-deflation shear rate data, derived from velocity and diameter measures, was used to calculate the area under the shear rate curve (SR_AUC_).

### Statistical Analysis

All data were analyzed using the Statistical Package for the Social Sciences (SPSS, version 16). Data are presented as mean±SD unless stated otherwise. Baseline parameters between testing days were compared by paired *t*-tests.

In a previous study from our laboratory, forearm IR reduced FMD with 2.6% with a SD of 3.7%. Assuming a correlation coefficient of 0.7 in our study, given the cross-over design, the expected SD of the effect of IR on FMD in our study therefore equals 2.85. With n = 26 subjects, we will be able to detect a difference of 1.65% with a power of 80% and a type I error probability of 5%, which is a relevant difference.

In order to evaluate the impact of IR on endothelial function (measured as FMD), and whether metformin can (partially) prevent endothelial IR, we employed a linear mixed model analysis and a two-way repeated measures ANOVA. Furthermore, according to a recent study by Atkinson *et al.*, inadequate scaling for FMD would be present if the upper confidence limit of the regression slope of the relationship between logarithmically transformed base diameter and peak diameter is less than one.[22] In such an event, FMD% is not an appropriate measure to estimate endothelial function. We checked our data for this phenomenon, and subsequently performed the allometric modelling solution proposed by Atkinson *et al.* [22] Subsequently, the FMD-values were re-analysed with a linear mixed model analysis with random factor subject and fixed factors IR (pre *versus* post), intervention (metformin *versus* no treatment), but also whether the type of intervention was associated with the different impact of IR on the change in FMD (i.e. interaction IR*intervention). We used a Kolmogorov-Smirnov test to demonstrate a normal distribution of our outcome measures (FMD% and allometrically scaled FMD; P>0.1). The level of statistical significance was set at 0.05.

## Results

Baseline characteristics are presented in Table 1. All values were within the normal range.

**Table 1:** **Baseline characteristics.**

Variable	Value
Age (yrs)	41.3±6.4
Body weight (kg)	74±13
Heigth (cm)	174±8
BMI (kg/m^2^)	24.2±2.9
Systolic blood pressure (mmHg)	120±9
Diastolic blood pressure (mmHg)	75±6
Creatinine (µmol/L)	73±13
MDRD-GFR (mL/min/1.73 m^2^)	85±7
Glucose (mmol/L)Cholesterol (mmol/L)	4.5±0.34.7±0.8

BMI; body mass index, MDRD; modification of diet in renal disease, GFR; glomerular filtration rate.

The metformin plasma concentration immediately before the experiment averaged 1357±588 ng/ml and were all within the therapeutic range (494–3237 ng/ml). Median plasma caffeine concentration was 0.12 (range 0–2.11) mg/dl, with 7 subjects >1 mg/dl. An overview of the FMD measurements is presented in table 2. There were no serious adverse events during the trial. At baseline, we found no differences in brachial artery characteristics (i.e. baseline diameter, time-to-peak diameter, and shear rate area-under-the-curve) between both testing days (Table 2). Baseline FMD% did not differ between metformin pretreatment and no pretreatment (6.9±3.6% and 6.1±3.5%, respectively, p = 0.27).

**Table 2:** **Brachial artery characteristics before and after ischemia-reperfusion (IR) when preceded by metformin pretreatment or no pretreatment (n = 26).**

	No pretreatment		Metformin			P-values		
	*Baseline*	*IR*	*Baseline*	*IR*	*IR*		*Metformin*	IR*Metformin
FMD (%)	6.1±3.5	4.3±2.7	6.9±3.6	4.4±3.3	<0.001		0.52	0.23
FMD (mm)	0.19±0.09	0.15±0.09	0.22±0.08	0.15±0.11	<0.001		0.60	0.09
Diameter (mm)	3.5±0.9	3.8±0.9	3.5±0.8	3.7±0.9	<0.001		0.52	0.15
Time-to-peak diameter (s)	49±16	54±25	54±17	48±23	0.94		0.92	0.17
SR_AUC_ (s, 10^3^)	30.2±11.3	21.8±11.7	28.4±11.2	21.1±9.5	<0.001		0.51	0.86

Data is presented as mean±SD.

FMD: flow mediated dilation; FMD (%) percent change to baseline; FMD (mm) absolute change to baseline; IR: ischemia-reperfusion; SR_AUC_: shear rate area under the curve.

**Figure 2:**
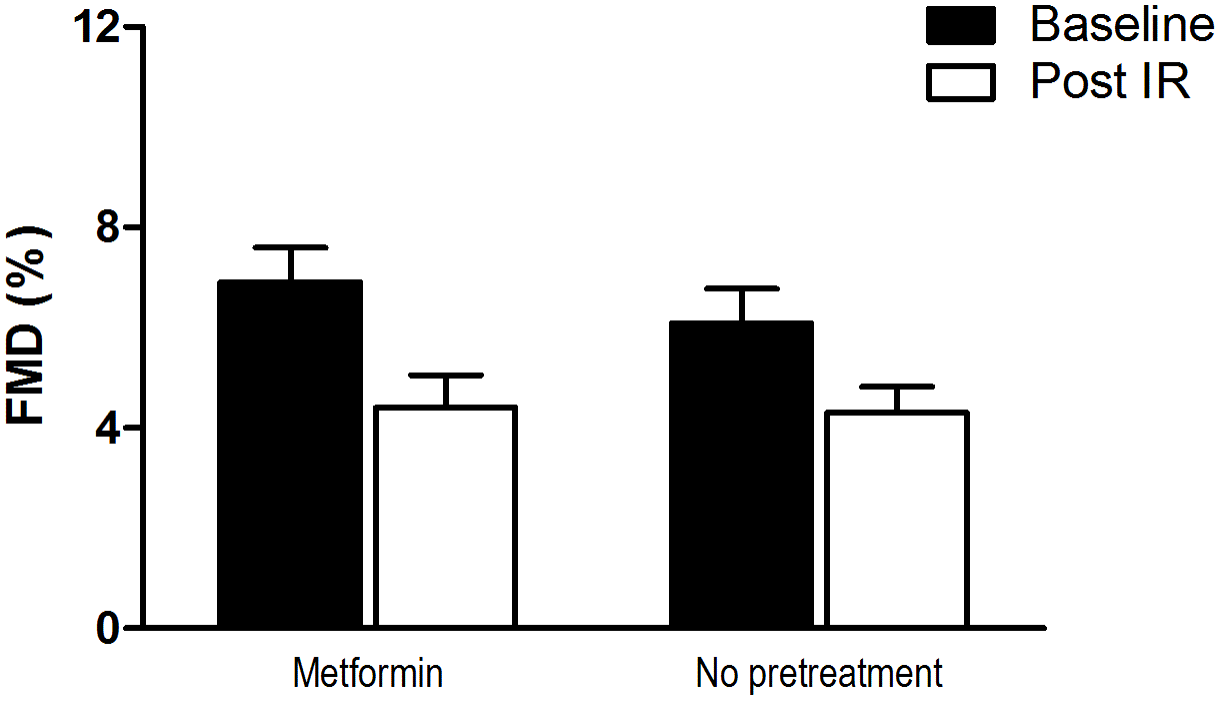
Brachial artery flow mediated dilation (presented as percentage change from baseline ±SE) before (black) and after (white) 20-minutes for forearm ischemia and 20-minutes reperfusion preceded by pretreatment with metformin or no pretreatment.

The IR protocol induced a significant increase in baseline brachial artery diameter and a decrease in shear rate stimulus that was not affected by metformin treatment (Table 2). Both in absence as well as in presence of metformin, brachial artery FMD% was significantly lower after forearm IR (4.4±3.3% and 4.3±2.7% respectively, p<0.01 in both conditions). A two-way repeated measures ANOVA revealed that metformin treatment did not affect the decrease in FMD by IR (Figure 2; p = 0.52). Subsequent linear mixed model analysis, in which we included baseline diameter and SR_AUC_ as covariates to correct for the changes in these parameters after IR, confirmed our primary finding, in that the decrease in FMD% after IR was not altered by 3-days of metformin intake. A limited number of subjects (n = 7) had a caffeine concentration >1 µg/ml. Exclusion of these subjects did not change our conclusion (data not shown).

## Discussion

Our current study is the first to investigate whether metformin limits IR-injury in humans *in vivo*. Using a well-validated model of forearm endothelial IR-injury in healthy middle-aged subjects, we demonstrated that a three day treatment with metformin does not protect against IR-induced endothelial dysfunction.

Our hypothesis that metformin ameliorates IR-injury is based on previous epidemiological studies and clinical trials in patient with diabetes and on preclinical animal studies. First, the United Kingdom Prospective Diabetes Study (UKPDS) reported that patients with type 2 diabetes mellitus who were treated with metformin had a significant lower cardiovascular mortality than patients treated with alternative glucose-lowering drugs, despite similar glycemic control.[2] Secondly, recent experimental studies in mice and rats showed that acute administration of metformin potently reduced myocardial infarct size. In diabetic and nondiabetic mice, administration of a single dose of metformin either before ischemia or at the moment of coronary reperfusion decreased final infarct size.[8]–[11] This cardioprotective effect appeared to be mediated by activation of AMPK and endothelial Nitric Oxide Synthase (NOS).[9] In addition, Yellon’s group reported that in diabetic and nondiabetic rat hearts, administration of metformin reduced infarct size.[8], [10], [23] In these studies, the cardioprotective effect was dependent on adenosine receptor stimulation and activation of important signalling molecules of the Reperfusion Injury Salvage Kinase (RISK) pathway. Finally, not only acute single-dose administration of metformin confers cardioprotection, but also chronic administration of metformin limits infarct size [23] and beneficially affects postinfarction myocardial remodelling.[11], [24] Based on these studies, we have recently proposed that metformin treatment is an attractive strategy to limit IR injury in patients suffering a myocardial infarction or patients undergoing cardiac surgery [7], [25].

To test our hypothesis, we used a well-validated model of IR-induced endothelial dysfunction in the forearm. Endothelial IR-injury is relevant for two reasons. First, myocardial ischemia and reperfusion not only inflicts direct injury to cardiomyocytes, but the endothelial cells are also highly susceptible to IR-injury.[12] Indeed, structural endothelial injury occurs during ischemia and reperfusion, which induces cell swelling and impairment of endothelial-dependent relaxation, which contributes to the so-called ‘no-reflow’phenomenon and impedes effective coronary reperfusion.[26], [27] Secondly, endothelial dysfunction is an early sign of cardiovascular disease and is associated with future cardiovascular events.[28]–[31] Importantly, endothelial dysfunction is associated with a worse outcome in several clinical settings.[32]–[35] Thus, IR injury to coronary endothelium could contribute to the increased risk of recurrent atherothrombosis as observed in patients who present with an acute coronary event.

Several previous studies have reported that twenty minutes of forearm ischemia impairs subsequent flow mediated dilation (FMD) of the brachial artery.[13], [14] Indeed, this finding was confirmed in our current study, in which post-IR FMD was 36% lower than baseline FMD. This observation of a lower FMD, even after correction for the potential influence of changes in diameter and the eliciting shear rate stimulus, is in agreement with the concept that IR causes endothelial dysfunction. Subsequent studies demonstrated that this IR-induced endothelial dysfunction can be prevented by ischemic preconditioning, postconditioning, and remote conditioning.[13], [14], [36], [37] Also, statins significantly reduced endothelial IR-injury in this model [38].

In contrast to the preclinical studies, we did not observe any protective effect of metformin against endothelial IR-injury in our study. There are several potential explanations for this discrepancy. First, murine models of IR-injury might not reflect the human situation. In this regard, many interventions that are promising in animal models do not appear to be effective in clinical trials.[39] In the field of ischemic stroke, only two of approximately 500 neuroprotective strategies that were beneficial in animal models, improved outcome in patients.[39] This translational failure can be due to differences between animal and human (patho)physiology, due to methodological flaws in animal studies, or due to shortcomings of the clinical trial. In the case of metformin-induced cardioprotection, the evidence is rather strong, with cardioprotection shown in mice and rats, with or without diabetes, and with acute as well as chronic administration. The design of our current study is also robust and uses a well-validated model of endothelial IR-injury. Although the study was not blinded, we used a PROBE design, which is a well-accepted design for this kind of studies.

A second explanation could relate to the duration and dose of metformin pretreatment. Interestingly, in several patient groups, including patients with type 1 diabetes and patients with polycystic ovarian syndrome, long-term administration of metformin improves endothelial function, measured with FMD.[40], [41] In our study, however, a three day treatment with metformin did not improve baseline FMD. Animal studies on the cardioprotective effect of metformin, however, have demonstrated that either an acute single dose administration of metformin[9], [10] as well as chronic administration of metformin confer cardioprotection.[23] The dose of metformin used in these preclinical studies was comparable to or even considerably lower than the dose used to treat patients with diabetes in clinical practice.[9], [10] Based on these studies, we used a dose of 3dd500 mg, which is a dose often used to treat patients in clinical practice. Subjects were pretreated for three days to ensure an effective steady-state plasma concentration and to enable translation of our results to daily clinical practice. The last dose of metformin was taken 3 hours prior to the ischemic episode, which allows for a maximum plasma concentration of metformin at the moment of forearm ischemia. Indeed, the circulating plasma concentration of metformin immediately before the experiment was comparable to the previous animal studies. In our study,

A third potential explanation for the discrepancy between our results and results from previous animal studies is that the mechanism of endothelial IR-injury might differ from IR-injury in cardiomyocytes. However, most strategies that limit myocardial infarct size in animal models also conferred protection against endothelial IR-injury, although some studies could not observe protection [13], [34], [36], [37].

Fourthly, the findings in the brachial artery may not be representative for the coronary circulation. However, previous studies have reported a good correlation between endothelial responses to flow and vasoactive substances between the brachial and coronary arteries [42], [43].

In conclusion, we can state that short-term metformin pretreatment does not protect against endothelial dysfunction induced by ischemia-reperfusion in healthy middle aged subject. However, whether these results predict the effect of metformin on myocardial IR-injury and can be extrapolated to subjects with a history of cardiovascular disease or diabetes mellitus is a matter of debate. It is important to realize that many comorbidities, including diabetes mellitus, and comedications can affect the tolerance against ischemia-reperfusion and the efficacy of cardioprotective strategies. Therefore, with interest we await further studies on the effect of metformin on myocardial injury in patients with a myocardial infarction and patients undergoing coronary artery bypass grafting (NCT01217307 and NCT01438723 respectively).

### Clinical Perspectives

In patients with diabetes, treatment with metformin is associated with an improved cardiovascular outcome. In contrast to previous studies in animals, we observed no protective effect of a short term treatment with metformin on forearm IR-induced endothelial dysfunction in healthy individuals. Further studies are needed to clarify what mechanism underlies the cardiovascular benefit of metformin treatment.

## Supporting Information

Checklist S1 CONSORT Checklist.
(DOCX)

Protocol S1 Trial Protocol.
(PDF)
