# Supplementary material for: Impact of Metformin on Endothelial Ischemia-Reperfusion Injury in Humans In Vivo: A Prospective Randomized Open, Blinded-Endpoint Study
Source: PLoS One. 2014 Apr 22;9(4):e96062. doi: 10.1371/journal.pone.0096062 (PMC3996005; doi:10.1371/journal.pone.0096062)
Supplement: Protocol S1 — Trial Protocol. (PDF) [file pone.0096062.s002.pdf]

Can metformin prevent endothelial ischemia and reperfusion injury?

The Metformin-FMD trial

(February 2012, version 3)

PROTOCOL TITLE "Can metformin prevent endothelial ischemia and reperfusion injury: The Metformin-FMD trial"

**Protocol ID**	**Metformin-FMD 001**
**Short title**	**The Metformin-FMD trial**
**Version**	**3**
**Date**	**February 2012**
**Study Sites**	**Radboud University Nijmegen Medical Centre (RUNMC)**
**Coordinating investigator**	**S. El Messaoudi, MD**<br>**149 Dept. of Pharmacology-Toxicology**<br>**RUNMC**
**Principal investigator RUNMC**<br>**(in Dutch: hoofdonderzoeker/uitvoerder**	**N.P. Riksen, MD, PhD**<br>**149 Dept. of Pharmacology-Toxicology**<br>**RUNMC**<br><br>**G.A. Rongen, MD, PhD**<br>**149 Dept. of Pharmacology-Toxicology**<br>**RUNMC**<br><br>**D.H. Thijssen, PhD**<br>**143 Dept. of Physiology**<br>**RUMC**
**Sponsor**	**P. Smits, MD, PhD, Head of Department**<br>**149 Dept. of Pharmacology-Toxicology**<br>**RUNMC**

**Independent physician(s)**	**C. Tack, MD, PhD**<br>**463 Dept. of Internal Medicine**<br>**RUNMC**
**Laboratory sites**	**Dept. Clinical Biochemistry**<br>**RUNMC**
**Pharmacy**	**Dept. Clinical Pharmacy**<br>**RUNMC**

PROTOCOL SIGNATURE SHEET

Name	Signature	Date
**Head of Department:**<br><br>**P. Smits, MD, PhD, internist-clinical pharmacologist**	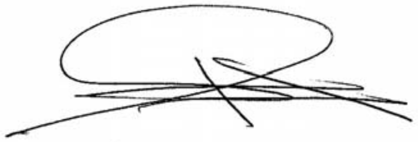	
**Principal Investigator:**<br>**G.A. Rongen, MD, PhD**<br>**RUNMC**<br><br>**N.P. Riksen, MD, PhD**<br>**RUNMC**<br><br>**D.H. Thijssen, PhD**<br>**143 Dept. of Physiology**<br>**RUMC**		
**Coordinating Investigator**<br>**S. El Messaoudi**		

TABLE OF CONTENTS

1. INTRODUCTION AND RATIONALE 8
2. OBJECTIVES 10
3. STUDY DESIGN 11
4. STUDY POPULATION 13
   - 4.1 Population (base) 13
   - 4.2 Inclusion criteria 13
   - 4.3 Exclusion criteria 13
   - 4.4 Sample size calculation 13
5. TREATMENT OF SUBJECTS .... **Fout! Bladwijzer niet gedefinieerd.**
   - 5.1 Investigational product/treatment .... **Fout! Bladwijzer niet gedefinieerd.**
   - 5.2 Use of co-intervention (if applicable) .... **Fout! Bladwijzer niet gedefinieerd.**
   - 5.3 Escape medication (if applicable) .... **Fout! Bladwijzer niet gedefinieerd.**
6. INVESTIGATIONAL MEDICINAL PRODUCT 15
   - 6.1 Name and description of investigational medicinal product 16
   - 6.2 Summary of findings from non-clinical studies 16
   - 6.3 Summary of findings from clinical studies 16
   - 6.4 Summary of known and potential risks and benefits 16
   - 6.5 Description and justification of route of administration and dosage 17
   - 6.6 Dosages, dosage modifications and method of administration 17
   - 6.7 Preparation and labelling of Investigational Medicinal Product 17
   - 6.8 Drug accountability 17
7. METHODS 19
   - 7.1 Study parameters/endpoints 19
     - 7.1.1 Main study parameter/endpoint 19
     - 7.1.2 Secondary study parameters/endpoints (if applicable) 19
     - 7.1.3 Other study parameters (if applicable) 19
   - 7.2 Randomisation, blinding and treatment allocation 19
   - 7.3 Study procedures 19
   - 7.4 Withdrawal of individual subjects 20
     - 7.4.1 Specific criteria for withdrawal (if applicable) 20
   - 7.5 Replacement of individual subjects after withdrawal 20
   - 7.6 Follow-up of subjects withdrawn from treatment 20
   - 7.7 Premature termination of the study 20
8. SAFETY REPORTING 21
   - 8.1 Section 10 WMO event 21
   - 8.2 Adverse and serious adverse events 21
     - 8.2.1 Suspected unexpected serious adverse reactions (SUSAR) 22
     - 8.2.2 Annual safety report 23
   - 8.3 Follow-up of adverse events 23
   - 8.4 Data Safety Monitoring Board (DSMB) 23
9. STATISTICAL ANALYSIS 24

9.1 Descriptive statistics 24
9.2 Univariate analysis 24
9.3 Multivariate analysis 24
9.4 Interim analysis (if applicable) 24
10. ETHICAL CONSIDERATIONS 25
10.1 Regulation statement 25
10.2 Recruitment and consent 25
10.3 Objection by minors or incapacitated subjects (if applicable) 25
10.4 Benefits and risks assessment, group relatedness 25
10.5 Compensation for injury 25
10.6 Incentives (if applicable) 26
11. ADMINISTRATIVE ASPECTS AND PUBLICATION 27
11.1 Handling and storage of data and documents 27
11.2 Amendments 27
11.3 Annual progress report 28
11.4 End of study report 28
11.5 Public disclosure and publication policy 28
12. REFERENCES 29

LIST OF ABBREVIATIONS AND RELEVANT DEFINITIONS

**ABR**	**ABR form, General Assessment and Registration form, is the application form that is required for submission to the accredited Ethics Committee (In Dutch, ABR = Algemene Beoordeling en Registratie)**
**AE**	**Adverse Event**
**AR**	**Adverse Reaction**
**CA**	**Competent Authority**
**CCMO**	**Central Committee on Research Involving Human Subjects; in Dutch: Centrale Commissie Mensgebonden Onderzoek**
**CV**	**Curriculum Vitae**
**DSMB**	**Data Safety Monitoring Board**
**EU**	**European Union**
**EudraCT**	**European drug regulatory affairs Clinical Trials**
**GCP**	**Good Clinical Practice**
**IB**	**Investigator's Brochure**
**IC**	**Informed Consent**
**IMP**	**Investigational Medicinal Product**
**IMPD**	**Investigational Medicinal Product Dossier**
**METC**	**Medical research ethics committee (MREC); in Dutch: medisch ethische toetsing commissie (METC)**
**(S)AE**	**(Serious) Adverse Event**
**SPC**	**Summary of Product Characteristics (in Dutch: officiële productinfomatie IB1-tekst)**
**Sponsor**	**The sponsor is the party that commissions the organisation or performance of the research, for example a pharmaceutical company, academic hospital, scientific organisation or investigator. A party that provides funding for a study but does not commission it is not regarded as the sponsor, but referred to as a subsidising party.**
**SUSAR**	**Suspected Unexpected Serious Adverse Reaction**
**Wbp**	**Personal Data Protection Act (in Dutch: Wet Bescherming Persoonsgevens)**
**WMO**	**Medical Research Involving Human Subjects Act (in Dutch: Wet Medisch-wetenschappelijk Onderzoek met Mensen**

SUMMARY

Rationale: In acute myocardial infarction early restoration of coronary blood flow is the most effective strategy to limit infarct-size. Paradoxically, reperfusion itself also aggravates myocardial injury and contributes to final infarct size, a process termed ‘reperfusion injury’. Ischemia and reperfusion (IR)-induced endothelial dysfunction seems to play a pivotal role in this process, resulting in vasoconstriction and reduced blood flow to the already ischemic tissue. Recently, it has been shown that the glucose-lowering drug metformin is able to limit IR-injury in murine models of myocardial infarction, probably by increased formation of the endogenous nucleoside adenosine. In the current research proposal, we aim to translate this finding to the human in vivo situation, using flow-mediated dilation (FMD) of the brachial artery as a well-validated model of (endothelial) IR-injury.

Objective: To study the protective effect of pre-treatment with metformin on flow mediated dilation after 20 minutes ischemia and 20 minutes reperfusion. If metformin treatment indeed limits endothelial IR-injury, a second, unblinded study will be performed in the same subjects to investigate whether this is mediated by adenosine receptor stimulation.

Study design: Randomized, single blind, , cross over clinical trial.

Study population: Healthy volunteers (n= 26 for both studies), age 30-50 years.

Intervention: *Main study (metformin FMD trial):* Pre-treatment with metformin 500 mg three times a day for 3 days or no medication. *Second study:* Pre-treatment with metformin 500 mg three times a day for 3 days or no medication, combined with caffeine infusion preceding FMD measurement.

Main study parameters/endpoints: Difference in flow mediated dilation before and after 20 minutes of ischemia.

Nature and extent of the burden and risks associated with participation, benefit and group relatedness: Healthy volunteers will be treated with metformin (500mg three times a day) for 3 days. Short-term treatment with metformin is not expected to induce serious side-effects. Monotherapy with metformin does not induce hypoglycemia. Metformin can give some gastrointestinal discomfort in the first days of treatment, but this is mild and self-limiting. Lactic acidosis is a rare, but serious, complication of metformin, which can occur in the setting of shock/hypoxia/renal failure in combination with metformin accumulation. This is therefore not to be expected in case of healthy volunteers. The participants will not benefit directly from participating in this study, but if our hypothesis proves to be correct, these findings will help to optimize future medical care of patients suffering from (myocardial) ischemia/infarction.

1. INTRODUCTION AND RATIONALE

In patients with an acute myocardial infarction early restoration of coronary blood flow is the most effective strategy to limit infarct-size. Paradoxically, reperfusion itself also aggravates myocardial injury and contributes to final infarct-size, a phenomenon that has been termed 'reperfusion injury'.[1] Ischemia and reperfusion (IR)-induced endothelial dysfunction seems to play a pivotal role in this process, where impairment in endothelium-dependent vasodilatation and an exaggerated response to endothelium-dependent vasoconstrictors result in vasoconstriction and reduced blood flow to ischemic areas.[2] Based on the high residual morbidity and mortality in patients suffering a myocardial infarction, novel strategies to reduce this injury are urgently needed.

In an effort to limit IR injury, a number of mechanisms that may display cardioprotective properties have been identified. An important mechanism involves activation of adenosine monophosphate-activated protein kinase (AMPK). Immediately after the onset of myocardial ischemia, AMPK is activated by phosphorylation, and remains activated for more than 24 hours following reperfusion[3]. The antihyperglycemic drug metformin has been suggested to activate AMPK and has recently been found to exhibit cardioprotective properties in murine models of myocardial infarction.[3-6] Metformin activates AMPK by increasing the intracellular AMP concentration, probably by inhibiting complex 1 of the mitochondrial respiratory chain.[7] We hypothesized that the increase in intracellular AMP also facilitates the formation of the endogenous purine adenosine.[5] Since adenosine receptor stimulation is known to increase tolerance against IR, this mechanism may well contribute to the cardioprotective effect of metformin. Indeed, the cardioprotective effect of metformin appeared to be completely abolished by a nonselective adenosine receptor antagonist in a rat infarction model.[5]

In the current study, we aim to translate the promising prospects of metformin to the human in vivo situation. First, we want to conduct an experiment in which healthy volunteers will be treated with or without metformin prior to an IR event to study whether metformin can also limit IR-injury in humans in vivo. As a model for IR-injury of the endothelium, we will use flow-mediated dilatation (FMD) of the brachial artery after a period of forearm ischemia and reperfusion. If proven effective, a second experiment will be conducted in which the adenosine receptor antagonist caffeine is co-administered with metformin to study whether the protective effect is indeed mediated by adenosine receptor stimulation. Flow mediated dilation represents a largely NO-mediated[8] endothelium-dependent dilation and has been shown to be decreased after forearm

ischemia and reperfusion, indicating IR-injury of the endothelial lining. [9, 10] Previous studies have validated FMD as a model for endothelial IR-injury, and showed that this reduction in FMD after ischemia and reperfusion can be prevented by several interventions that are known to prevent ischemia reperfusion injury (e.g. ischemic preconditioning[9], remote preconditioning[11], ischemic postconditioning[10], bradykinin administration[12], NO administration[13] and statins[14, 15]). FMD of the brachial artery as a measure of endothelial (dys)function is therefore a well validated model to study effects of possible protective strategies and perform mechanistic experiments on IR-injury in humans in vivo.

2. OBJECTIVES

Primary Objective:

Metformin-FMD trial:
To study the effect of oral pretreatment with metformin (500 mg three times a day for 3 days) on flow mediated dilation of the brachial artery after 20 minutes of forearm ischemia and 20 minutes reperfusion in healthy volunteers.

Control study:
To study whether caffeine, a non-selective competitive antagonist of adenosine receptors, attenuates the protective effect of metformin on flow mediated dilation after 20 minutes ischemia and 20 minutes reperfusion in healthy volunteers.

Secondary Objective(s): not applicable.

3. STUDY DESIGN

After written informed consent all healthy volunteers will undergo medical screening consisting of a short interview, physical examination, laboratory evaluation (glucose, cholesterol, kreatinine) and EKG. Females using oral contraceptives will be asked to continue their contraceptive throughout the study to maintain stable hormone levels. Females not on oral contraceptives will be measured at identical times in their menstrual cycle.[16, 17] As this is a cross-over model and subjects are their own control, potential differences in baseline FMD are not to be expected to influence results. For both trials, volunteers eligible for trial participation will receive study medication and instructions how to use it. The first study is a randomized, single-blinded cross-over study. 26 participants wil be recruited and half of the participants will be pretreated with metformin 500 mg three times a day for 3 days, to ensure a steady state plasma concentration, whereas the other half will receive no medication The two experiments will be separated by approximately two weeks to prevent any cross-over effect of metformin. The last dose of metformin will be given approximately 3 hours before the experiments. The FMD measurements will be performed, by an operator blinded for pretreatment after an overnight fast and 24 hours of caffeine abstinence (since caffeine is an adenosine receptor antagonist). Also, the participants have to refrain from strenuous exercise for at least 24 hours before the experiment, as this potentially act as a preconditioning stimulus. Experiments will be performed in a temperature-controlled room (22° C) and according to recent guidelines for assessment of the FMD.[18] Measurements will always be performed at the same time of day to prevent diurnal variation in the FMD response.[19]

When metformin displays a protective effect on endothelial function in this RCT, a second experiment will be conducted in the same volunteers to further elucidate the underlying mechanism. For this experiment the design will be identical to the metformin-FMD trial, with the exception that the subjects will all be pretreated with caffeine (4 mg/kg intravenously over 10 minutes) prior to the FMD measurement. This dose has previously been shown to completely prevent the protective effect of ischemic preconditioning which is also mediated by adenosine receptor stimulation in another human experimental model of forearm ischemia-reperfusion injury [20]. This study will provide the answer to the question whether the protective effect of metformin is mediated by adenosine receptor stimulation by comparing the results of the FMD measurements with and without concomitant administration of caffeine. In addition, this study will enable us to exclude that any effect of caffeine on metformin-induced protection is due to a direct effect of caffeine on FMD or on the effect of IR on FMD: this will be studied by comparing the FMD measurements after caffeine administration in the placebo-arms.

FMD measurement before and after 20 minutes ischemia

Participants will rest in the supine position in a temperature controlled room (22 ^{o}C) for at least 15-minutes to facilitate baseline assessment of heart rate and blood flow. To examine brachial artery FMD, the right arm will be extended and positioned at an angle of ~80° from the torso. A rapid inflation and deflation pneumatic cuff (D.E. Hokanson, Bellevue, WA) will be positioned on the forearm immediately distal to the olecranon process to enable induction of forearm ischemia.[18] A 10-MHz multi-frequency linear array probe attached to a high resolution ultrasound machine (T3000; Terason, Burlington, MA) will be used to image the brachial arteries in the distal 1/3rd of the upper arm. When an optimal image is obtained, the probe will be held stable and the ultrasound parameters are set to optimize the longitudinal, B-mode images of lumen-arterial wall interface. Continuous Doppler velocity assessment will be simultaneously obtained using the ultrasound machine, and will be collected using the lowest possible insonation angle (always <60°), which will not vary during each study. Baseline images are recorded for 1 minute. The forearm cuff will be then inflated (>200 mmHg) for 5-minutes. Diameter and blood flow recordings will be resumed 30 seconds prior to cuff deflation and continued for 3 minutes thereafter. The baseline FMD measurement will be followed by the experimental IR-injury. The pneumatic cuff will be placed proximally around the upper arm and inflated to 200mmHg to apply 20 minutes of ischemia followed by cuff deflation. After 20 minutes of reperfusion, the procedures for FMD assessment as described above are repeated by the same experienced sonographer.

Caffeine experiment:

The FMD measurements will be preceded by a caffeine-infusion (4 mg/kg body weight intravenously over 10 minutes) 15 minutes before the IR intervention.

In both studies, blood will be drawn immediately before the first FMD measurement for the determination of the plasma metformin and caffeine concentration.

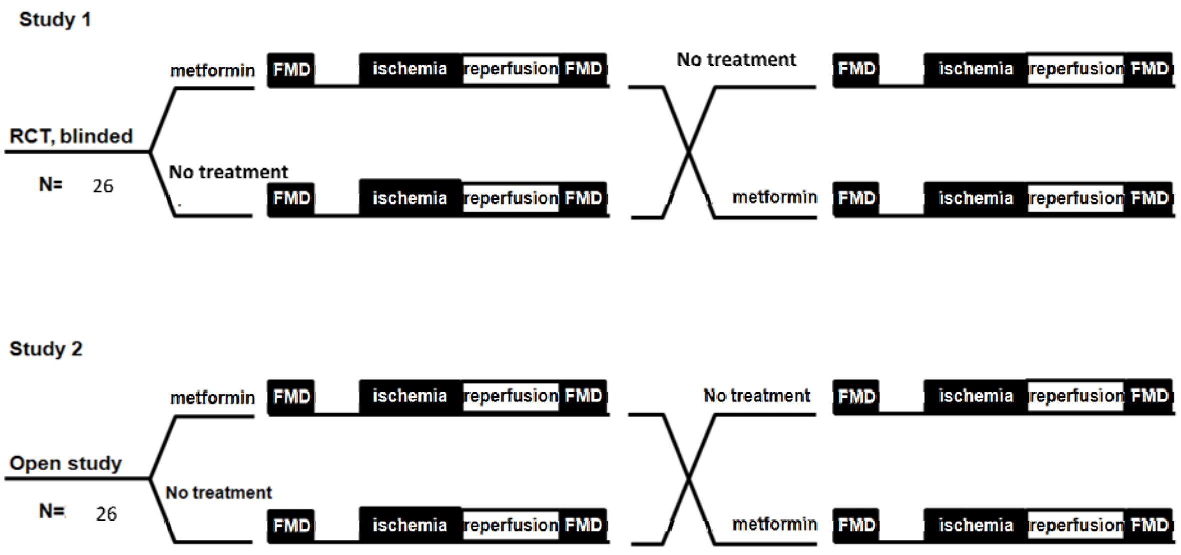

4. STUDY POPULATION

4.1 Population (base)

- Healthy volunteers (m/f)

4.2 Inclusion criteria

- Age 30-50 years
- Written informed consent

4.3 Exclusion criteria

- Smoking
- Hypertension (in supine position: systolic BP > 140 mmgHg, diastolic BP > 90 mmHg)
- Hyperlipidaemia (fasting total cholesterol > 5.5 mmol/L or random > 6.5 mmol/L)
- Diabetes Mellitus (fasting glucose > 7.0 mmol/L or random glucose > 11.0 mmol/L)
- History of any cardiovascular disease
- Concomitant use of medication
- Renal dysfunction (MDRD < 60 ml/min)
- Professional athletes

4.4 Sample size calculation

A recent study with a similar experimental design from our research group[21] has reported that the FMD averages 7.7% before, and 5.1% after forearm IR. Therefore, the effect of IR

on FMD is 2.6% with a SD of 3.7%. However, this study did not have a cross-over design. A previous cross-over study from Thijssen et al. (unpublished results) has reported a Spearman correlation coefficient of 0.53 between the two paired FMD measurements, but in this study one of the FMD measurements was preceded by ischemic preconditioning. Thus, the correlation coefficient with two similar measurements of FMD is most likely stronger than 0.53. Assuming a correlation coefficient of 0.7, the expected SD of the effect of IR on FMD in our study therefore equals 2.85. With n = 26 subjects, we will be able to detect a difference of 1.65% with a power of 80% and a type I error probability of 5%, which is a relevant difference.

5. TREATMENT OF SUBJECTS

5.1 Investigational product/treatment

In the first study, patients will be randomized to either metformin 500mg three times a day for 3 days before experiments. Participants are not allowed to drink caffeine containing beverages starting 24 hours prior to measurements.

In the second study, the subjects will also be pre-treated with metformin or no medication before each of the two experiments, and will all receive caffeine (4 mg/kg iv in ten minutes) 15 minutes before the experiment.

5.2 Use of co-intervention (if applicable)

Not applicable.

5.3 Escape medication (if applicable)

Not applicable.

6. INVESTIGATIONAL MEDICINAL PRODUCT

6.1 Name and description of investigational medicinal product(s)

Metformin is an antihyperglycaemic drug that reduces plasma glucose levels in patients with diabetes mellitus by improving insulin sensitivity, which results in a reduced hepatic glucose production and improved peripheral glucose uptake. Metformin is registered in the Netherlands as a first-line treatment of patients with type 2 diabetes mellitus.

Caffeine is the most widely consumed drug worldwide, and acts as a nonselective adenosine receptor antagonist. The dose of 4 mg/kg is equivalent to the dose in 2-3 cups of regular coffee.

6.2 Summary of findings from non-clinical studies

In murine models of myocardial infarction, the administration of metformin (in a dose comparable to the dose normally used to treat patients with type 2 diabetes mellitus), either before ischemia or immediately at the moment of reperfusion, profoundly reduced myocardial infarction with approximately 50% [3, 5]

6.3 Summary of findings from clinical studies

Large epidemiological trials have reported that patients with type 2 diabetes mellitus who were treated with metformin had a lower total and cardiovascular mortality than patients treated with alternative glucose-lowering drugs, despite similar glycaemic control.[22] In addition, a retrospective analysis of the Prevention of Restenosis with Tranilast and its Outcomes Trial (PRESTO) showed that the use of metformin reduced death and myocardial infarction in diabetics undergoing coronary interventions, compared with diabetic patients treated with alternative glucose-lowering drugs.[23]

6.4 Summary of known and potential risks and benefits

Metformin is registered as a first-line treatment of patients with type 2 diabetes mellitus, and is the most widely prescribed antihyperglycemic drug worldwide. As mentioned previously, large prospective epidemiological studies have shown that treatment with metformin reduces total and cardiovascular mortality compared to alternative antihyperglycemic drugs. The most common side-effects are gastro-intestinal complaints, including nausea, bloating, abdominal pain and diarrhea. These complaints are usually mild and self-limiting. Lactic acidosis is a rare, but serious complication of metformin use, with an estimated incidence of 1-5 cases per 100.000 patient years[24, 25], occurring mainly during metformin accumulation in the setting of chronic metformin use. In addition, it has

been suggested that metformin-induced lactic acidosis only occurs in the setting of other predisposing factors for lactic acidosis, such as renal dysfunction, hepatic dysfunction, of tissue hypoxia. In the absence of these alternative risk factors (i.e. in the case of acute overdose), metformin only induces life-threatening lactic acidosis in the presence of very high plasma concentrations of metformin[26]. Lactic acidosis is therefore not to be expected in case of healthy volunteers.

Caffeine will be given in a dose comparable to 2-3 cups of regular coffee. In this dose, the blood pressure will increase modestly [20] We have used this dose in many previous studies as a tool to effectively block adenosine receptors, and this dose is not expected to give any unwanted side-effects[20, 27].

6.5 Description and justification of route of administration and dosage

The subjects will be treated with 500 mg of metformin three times a day for 3 days before experiments. To ensure a peak plasma metformin concentration at the moment of experiment, the last dose will de administered approximately 3 hours before start of experiment (T_{max} of metformin is 3 hours).

Caffeine will be given in a dose of 4 mg/kg intravenously over ten minutes. We have previously shown that this dose results in a plasma concentration that is comparable to the concentration after drinking 2-3 cups of regular coffee, and that this concentration effectively blocks the adenosine receptors involved in protection against IR-injury[20]

6.6 Dosages, dosage modifications and method of administration

Metformin tablets of 500 mg three times a day during 3 days.

6.7 Preparation and labelling of Investigational Medicinal Product

Tablets containing metformin 500 mg will be provided by the department of Clinical Pharmacy of the Radboud University Nijmegen Medical Centre according to GMP-standards. These medicinal products will be stocked at the department of Clinical Physiology at the RUNMC at a controlled temperature (15-25° C).

Caffeine will be obtained from the department of Clinical Pharmacy (ampuls of 5 ml containing 10 mg/ml) and dissolved in 100 ml of saline.

6.8 Drug accountability

The department of Clinical Pharmacy of the Radboud University Medical Centre will provide the metformin used in this trial. The products are transported to the department of Clinical Physiology at the RUNMC and stored there under GMP conditions. Compliance to the treatment with metformin will be checked by pill counting. The completed drug accountability of all involved medicinal products will be documented by the investigator on specially designed drug accountability form.

7. METHODS

7.1 Study parameters/endpoints

7.1.1 Main study parameter/endpoint

Difference in flow mediated dilation before and after 20 minutes of forearm ischemia and reperfusion.

7.1.2 Secondary study parameters/endpoints (if applicable)

Not applicable.

7.1.3 Other study parameters (if applicable)

Baseline screening (BMI, blood pressure, glucose, renal function).

7.2 Randomisation, blinding and treatment allocation

The study is a single blinded randomized cross-over trial. The code will be broken only after all patients have completed the protocol. However, in case of any serious adverse event, if necessary, the individual code for the concerning participant will be broken, to enable and facilitate possible medical treatment.

7.3 Study procedures

Enrollment check:

At entry of this study all volunteers will undergo an enrolment check consisting of a medical interview and physical examination performed by a qualified physician. EKG and venous puncture will be performed for some screening lab tests (total cholesterol, renal function (creatinine) and glucose). Just before the FMD measurement, a venous puncture will be performed for the determination of caffeine and metformin concentration.

Medication:

In both studies, all volunteers are treated with either metformin (500 mg tid for 3 days) until the day of experiment.
In the second part of the study all subjects will receive caffeine (4 mg/kg intravenously infused over 10 minutes) immediately before the first FMD measurement.

FMD measurement:

As described in section 3, study design.

7.4 Withdrawal of individual subjects

Subjects can leave the study at any time for any reason if they wish to do so without any consequences. The investigator can decide to withdraw a subject from the study for urgent medical reasons.

7.4.1 Specific criteria for withdrawal (if applicable)

Not applicable

7.5 Replacement of individual subjects after withdrawal

If a participant decides to withdraw from the trial, replacement will be recruited in the same fashion the original participants were recruited.

7.6 Follow-up of subjects withdrawn from treatment

A participant who decides to withdraw from the trial will be invited to an exit-interview concerning their withdrawal.

7.7 Premature termination of the study

In case there of any unexpected and unforeseen complication or side-effect that is related to our study, continuation of the protocol will be discussed with responsible investigators and local ethics committee.

8. SAFETY REPORTING

8.1 Section 10 WMO event

In accordance to section 10, subsection 1, of the WMO, the investigator will inform the patients and the reviewing accredited METC if anything occurs, on the basis of which it appears that the disadvantages of participation may be significantly greater than was foreseen in the research proposal. The study will be suspended pending further review by the accredited METC, except insofar as suspension would jeopardise the patients' health. The investigator will take care that all patients are kept informed.

8.2 Adverse and serious adverse events

Adverse events are defined as any undesirable experience occurring to a subject during the study, whether or not considered related to the investigational drug. All adverse events reported spontaneously by the subject or observed by the investigator or his staff will be recorded.

A serious adverse event is any untoward medical occurrence or effect that at any dose:

- results in death;
- is life threatening (at the time of the event);
- requires hospitalisation or prolongation of existing inpatients' hospitalisation;
- results in persistent or significant disability or incapacity;
- is a congenital anomaly or birth defect;
- is a new event of the trial likely to affect the safety of the subjects, such as an unexpected outcome of an adverse reaction, lack of efficacy of an IMP used for the treatment of a life threatening disease, major safety finding from a newly completed animal study, etc.

All serious adverse events will be reported to the sponsor of this trial: Prof. P. Smits, head of the department of Pharmacology and Toxicology. He delegates the appropriate handling of these events to the coordinating and principle investigators of this trial: Prof. dr. G. A. Rongen and Dr. N.P. Riksen, Further report of these adverse events will be performed according to GCP as outlined below.

All SAEs will be reported through the web portal *ToetsingOnline* to the accredited METC that approved the protocol, within 15 calendar days after the sponsor has first knowledge of the serious adverse reactions.

SAEs that result in death or are life threatening should be reported expedited. The expedited reporting will occur not later than 7 calendar days after the responsible investigator has first knowledge of the adverse reaction. This is for a preliminary report with another 8 days for completion of the report.

8.2.1 Suspected unexpected serious adverse reactions (SUSAR)

Adverse reactions are all untoward and unintended responses to an investigational product related to any dose administered.

Unexpected adverse reactions are adverse reactions, of which the nature, or severity, is not consistent with the applicable product information (e.g. Investigator's Brochure for an unapproved IMP or Summary of Product Characteristics (SPC) for an authorised medicinal product).

The sponsor will report expedited the following SUSARs through the web portal *ToetsingOnline* to the METC:

- SUSARs that have arisen in the clinical trial that was assessed by the METC;
- SUSARs that have arisen in other clinical trials of the same sponsor and with the same medicinal product, and that could have consequences for the safety of the subjects involved in the clinical trial that was assessed by the METC.

The remaining SUSARs are recorded in an overview list (line-listing) that will be submitted once every half year to the METC. This line-listing provides an overview of all SUSARs from the study medicine, accompanied by a brief report highlighting the main points of concern. The expedited reporting of SUSARs through the web portal ToetsingOnline is sufficient as notification to the competent authority.

The sponsor will report expedited all SUSARs to the competent authority, through the portal of the CCMO, who will subsequently report to the Medicine Evaluation Board and the competent authorities in other Member States. If a SAE or SUSAR occurs and there is the need to know whether a patient was treated with metformin or placebo, the pharmacist on call can be contacted (24 hours a day, 7 days a week) and he/she will be able to break the blinding code for that particular participant. The expedited reporting will occur not later than 15 calendar days after the sponsor has first knowledge of the adverse reactions. For fatal or life threatening cases the term will be maximal 7 days for a preliminary report with another 8 days for completion of the report.

8.2.2 Annual safety report

In addition to the expedited reporting of SUSARs, the sponsor will submit, once a year throughout the clinical trial, a safety report to the accredited METC, competent authority, Medicine Evaluation Board and competent authorities of the concerned Member States.

This safety report consists of:

- a list of all suspected (unexpected or expected) serious adverse reactions, along with an aggregated summary table of all reported serious adverse reactions, ordered by organ system, per study;
- a report concerning the safety of the subjects, consisting of a complete safety analysis and an evaluation of the balance between the efficacy and the harmfulness of the medicine under investigation.

8.3 Follow-up of adverse events

All adverse events will be followed until they have abated, or until a stable situation has been reached. Depending on the event, follow up may require additional tests or medical procedures as indicated, and/or referral to the general physician or a medical specialist.

8.4 Data Safety Monitoring Board (DSMB)

Not applicable.

9. STATISTICAL ANALYSIS

9.1 Descriptive statistics

Descriptive statistics will be presented as mean ±SD, unless otherwise specified.

9.2 Univariate analysis

A paired-sample t-test will be used to test whether metformin can attenuate the decrease in FMD after forearm IR. The same method will be used in the second study to explore the effect of caffeine on metformin-induced protection, and the effect of caffeine on FMD per se.

9.3 Multivariate analysis

Not applicable

9.4 Interim analysis (if applicable)

Not applicable

10. ETHICAL CONSIDERATIONS

10.1 Regulation statement

This study will be conducted according to the principles of the Declaration of Helsinki (version: April 2008) and in accordance with the Medical Research Involving Human Subjects Act (WMO) and other guidelines and regulations.

10.2 Recruitment and consent

Healthy volunteers will be recruited at the University Medical Centre Nijmegen. Informed consent will be achieved by a ‘participation information letter’ and potential participants will be given the opportunity to address their questions to the investigator. Participation is only possible after written informed consent. Please find the patient information letter and informed consent form as separate documents.

10.3 Objection by minors or incapacitated subjects (if applicable)

Not applicable

10.4 Benefits and risks assessment, group relatedness

This study will be executed at the Radboud University Nijmegen Medical Centre under close medical supervision. Treatment with metformin or caffeine, is not expected to harm the participants. Monotherapy with metformin does not induce hypoglycemia. Most common side effects of metformin are gastro-intestinal problems, which are mild and self limiting. The occurrence of lactic acidosis is very rare and not to be expected in healthy volunteers. The ischemia reperfusion protocol (20 minutes forearm ischemia) will not induce long term side effects or damage and is tolerated well by volunteers.

10.5 Compensation for injury

The sponsor/investigator has a liability insurance which is in accordance with article 7, subsection 6 of the WMO.

The sponsor (also) has an insurance which is in accordance with the legal requirements in the Netherlands (Article 7 WMO and the Measure regarding Compulsory Insurance for Clinical Research in Humans of 23th June 2003). This insurance provides cover for damage to research subjects through injury or death caused by the study.

1. € 450.000,-- (i.e. four hundred and fifty thousand Euro) for death or injury for each subject who participates in the Research;

2. € 3.500.000,-- (i.e. three million five hundred thousand Euro) for death or injury for all subjects who participate in the Research;
3. € 5.000.000,-- (i.e. five million Euro) for the total damage incurred by the organisation for all damage disclosed by scientific research for the Sponsor as 'verrichter' in the meaning of said Act in each year of insurance coverage.

The insurance applies to the damage that becomes apparent during the study or within 4 years after the end of the study.

10.6 Incentives (if applicable)

Volunteers will be paid 300 euro at completion of the protocol (i.e. four experiments) as a compensation. Should the volunteer decide to withdraw from the study before the last measurement, then the compensation will be proportional to the number of experiments that the subject has participated in (e.g. if the subjects withdraws after the first RCT, than 150 euro's will be paid).

11. ADMINISTRATIVE ASPECTS AND PUBLICATION

11.1 Handling and storage of data and documents

All research outcomes will be archived in the personal medical file of each participant. This medical file will never leave the Radboud University Nijmegen Medical Centre. This source document can only be viewed by trial monitors and investigators involved in trials for which subject's signed informed consent has been obtained. The following data will be archived in the source document: Copy of signed informed consent form, EKG, Subject study code, each visit date and all study outcomes (laboratory) and possible adverse events, (serious) adverse events. In addition to the source document, a case report form (CRF) will be completed. The CRF is anonymised (only contains subject initials and subject study code) and may leave the hospital for data management or monitoring purposes. A copy of this CRF remains at the RUNMC for 15 years.

The following data will be archived in the CRF: Subject code; date, all study outcomes (laboratory) and possible adverse events, (serious) adverse events. A subject code log will be archived in a locked site in the trial master file. Furthermore we will file the drug accountability forms in the trial master file together with the approved version of the protocol, correspondence with the METC and CVs of all involved investigators.

11.2 Amendments

A 'substantial amendment' is defined as an amendment to the terms of the METC application, or to the protocol or any other supporting documentation, that is likely to affect to a significant degree:

- the safety or physical or mental integrity of the subjects of the trial;
- the scientific value of the trial;
- the conduct or management of the trial; or
- the quality or safety of any intervention used in the trial.

All substantial amendments will be notified to the METC and to the competent authority.

Non-substantial amendments will not be notified to the accredited METC and the competent authority, but will be recorded and filed by the sponsor.

11.3 Annual progress report

The sponsor/investigator will submit a summary of the progress of the trial to the accredited METC once a year. Information will be provided on the date of inclusion of the first subject, numbers of subjects included and numbers of subjects that have completed the trial, serious adverse events/ serious adverse reactions, other problems, and amendments.

11.4 End of study report

The sponsor will notify the accredited METC and the competent authority of the end of the study within a period of 90 days. The end of the study is defined as the last patient's last visit.

In case the study is ended prematurely, the sponsor will notify the accredited METC and the competent authority within 15 days, including the reasons for the premature termination.

Within one year after the end of the study, the investigator/sponsor will submit a final study report with the results of the study, including any publications/abstracts of the study, to the accredited METC and the Competent Authority.

11.5 Public disclosure and publication policy

There are no limitations on public disclosure or publication whatsoever. Results will be submitted for publication to a peer reviewed medical journal as soon as possible. The protocol summary will be made public on www.clinicaltrials.gov.

12. REFERENCES

1. Yellon DM, Hausenloy DJ. Myocardial reperfusion injury. *N Engl J Med*. 2007;357:1121-1135
2. Moens AL, Claeys MJ, Timmermans JP, Vrints CJ. Myocardial ischemia/reperfusion-injury, a clinical view on a complex pathophysiological process. *Int J Cardiol*. 2005;100:179-190
3. Calvert JW, Gundewar S, Jha S, Greer JJ, Bestermann WH, Tian R, Lefer DJ. Acute metformin therapy confers cardioprotection against myocardial infarction via ampk-enos-mediated signaling. *Diabetes*. 2008;57:696-705
4. Bhamra GS, Hausenloy DJ, Davidson SM, Carr RD, Paiva M, Wynne AM, Mocanu MM, Yellon DM. Metformin protects the ischemic heart by the akt-mediated inhibition of mitochondrial permeability transition pore opening. *Basic Res Cardiol*. 2008;103:274-284
5. Paiva M, Riksen NP, Davidson SM, Hausenloy DJ, Monteiro P, Goncalves L, Providencia L, Rongen GA, Smits P, Mocanu MM, Yellon DM. Metformin prevents myocardial reperfusion injury by activating the adenosine receptor. *J Cardiovasc Pharmacol*. 2009;53:373-378
6. Gundewar S, Calvert JW, Jha S, Toedt-Pingel I, Ji SY, Nunez D, Ramachandran A, Anaya-Cisneros M, Tian R, Lefer DJ. Activation of amp-activated protein kinase by metformin improves left ventricular function and survival in heart failure. *Circ Res*. 2009;104:403-411
7. Zhang L, He H, Balschi JA. Metformin and phenformin activate amp-activated protein kinase in the heart by increasing cytosolic amp concentration. *Am J Physiol Heart Circ Physiol*. 2007;293:H457-466
8. Mullen MJ, Kharbanda RK, Cross J, Donald AE, Taylor M, Vallance P, Deanfield JE, MacAllister RJ. Heterogenous nature of flow-mediated dilatation in human conduit arteries in vivo: Relevance to endothelial dysfunction in hypercholesterolemia. *Circ Res*. 2001;88:145-151
9. Kharbanda RK, Peters M, Walton B, Kattenhorn M, Mullen M, Klein N, Vallance P, Deanfield J, MacAllister R. Ischemic preconditioning prevents endothelial injury and systemic neutrophil activation during ischemia-reperfusion in humans in vivo. *Circulation*. 2001;103:1624-1630
10. Loukogeorgakis SP, Williams R, Panagiotidou AT, Kolvekar SK, Donald A, Cole TJ, Yellon DM, Deanfield JE, MacAllister RJ. Transient limb ischemia induces remote preconditioning and remote postconditioning in humans by a k(atp)-channel dependent mechanism. *Circulation*. 2007;116:1386-1395
11. Loukogeorgakis SP, Panagiotidou AT, Broadhead MW, Donald A, Deanfield JE, MacAllister RJ. Remote ischemic preconditioning provides early and late protection

against endothelial ischemia-reperfusion injury in humans: Role of the autonomic nervous system. *J Am Coll Cardiol*. 2005;46:450-456
12. Liuba P, Batra S, Pesonen E, Werner O. Bradykinin preconditions postischemic arterial endothelial function in humans. *J Card Surg*. 2005;20:420-424
13. Gori T, Di Stolfo G, Sicuro S, Dragoni S, Lisi M, Forconi S, Parker JD. Nitroglycerin protects the endothelium from ischaemia and reperfusion: Human mechanistic insight. *Br J Clin Pharmacol*. 2007;64:145-150
14. Meijer P, Oyen WJ, Dekker D, van den Broek PH, Wouters CW, Boerman OC, Scheffer GJ, Smits P, Rongen GA. Rosuvastatin increases extracellular adenosine formation in humans in vivo: A new perspective on cardiovascular protection. *Arterioscler Thromb Vasc Biol*. 2009;29:963-968
15. Liuni A, Luca MC, Gori T, Parker JD. Rosuvastatin prevents conduit artery endothelial dysfunction induced by ischemia and reperfusion by a cyclooxygenase-2-dependent mechanism. *J Am Coll Cardiol*. 2010;55:1002-1006
16. Hashimoto M, Akishita M, Eto M, Ishikawa M, Kozaki K, Toba K, Sagara Y, Taketani Y, Orimo H, Ouchi Y. Modulation of endothelium-dependent flow-mediated dilatation of the brachial artery by sex and menstrual cycle. *Circulation*. 1995;92:3431-3435
17. Williams MR, Westerman RA, Kingwell BA, Paige J, Blombery PA, Sudhir K, Komesaroff PA. Variations in endothelial function and arterial compliance during the menstrual cycle. *J Clin Endocrinol Metab*. 2001;86:5389-5395
18. Thijssen DH, Black MA, Pyke KE, Padilla J, Atkinson G, Harris RA, Parker B, Widlansky ME, Tschakovsky ME, Green DJ. Assessment of flow-mediated dilation in humans: A methodological and physiological guideline. *Am J Physiol Heart Circ Physiol*. 2011;300:H2-12
19. Atkinson G, Jones H, Ainslie PN. Circadian variation in the circulatory responses to exercise: Relevance to the morning peaks in strokes and cardiac events. *Eur J Appl Physiol*. 2010;108:15-29
20. Riksen NP, Zhou Z, Oyen WJ, Jaspers R, Ramakers BP, Brouwer RM, Boerman OC, Steinmetz N, Smits P, Rongen GA. Caffeine prevents protection in two human models of ischemic preconditioning. *J Am Coll Cardiol*. 2006;48:700-707
21. Wouters CW, Wever KE, Bronckers I, Hopman MT, Smits P, Thijssen DH, Rongen GA. Short-term statin treatment does not prevent ischemia and reperfusion-induced endothelial dysfunction in humans. *J Cardiovasc Pharmacol*. 2011
22. Effect of intensive blood-glucose control with metformin on complications in overweight patients with type 2 diabetes (ukpds 34). Uk prospective diabetes study (ukpds) group. *Lancet*. 1998;352:854-865

23. Kao J, Tobis J, McClelland RL, Heaton MR, Davis BR, Holmes DR, Jr., Currier JW. Relation of metformin treatment to clinical events in diabetic patients undergoing percutaneous intervention. *Am J Cardiol*. 2004;93:1347-1350, A1345
24. Salpeter SR, Greyber E, Pasternak GA, Salpeter EE. Risk of fatal and nonfatal lactic acidosis with metformin use in type 2 diabetes mellitus. *Cochrane Database Syst Rev*. 2010:CD002967
25. Brown JB, Pedula K, Barzilay J, Herson MK, Latare P. Lactic acidosis rates in type 2 diabetes. *Diabetes Care*. 1998;21:1659-1663
26. Dell'Aglio DM, Perino LJ, Kazzi Z, Abramson J, Schwartz MD, Morgan BW. Acute metformin overdose: Examining serum ph, lactate level, and metformin concentrations in survivors versus nonsurvivors: A systematic review of the literature. *Ann Emerg Med*. 2009;54:818-823
27. Meijer P, Wouters CW, van den Broek PH, Scheffer GJ, Riksen NP, Smits P, Rongen GA. Dipyridamole enhances ischaemia-induced reactive hyperaemia by increased adenosine receptor stimulation. *Br J Pharmacol*. 2008;153:1169-1176
